# Supplementary material for: DMRforPairs: identifying Differentially Methylated Regions between unique samples using array based methylation profiles
Source: BMC Bioinformatics. 2014 May 15;15:141. doi: 10.1186/1471-2105-15-141 (PMC4046028; doi:10.1186/1471-2105-15-141)
Supplement: Additional file 2 — DMRforPairs output for the comparison of A431-MCF7 and NA17018-NA17105. Please start from the HTML files in each folder. Available via the BMC Bioinformatics website. [file 1471-2105-15-141-S2.zip › 1394847754114233_MOESM2_ESM/A431_MCF7/figures/15107.pdf]

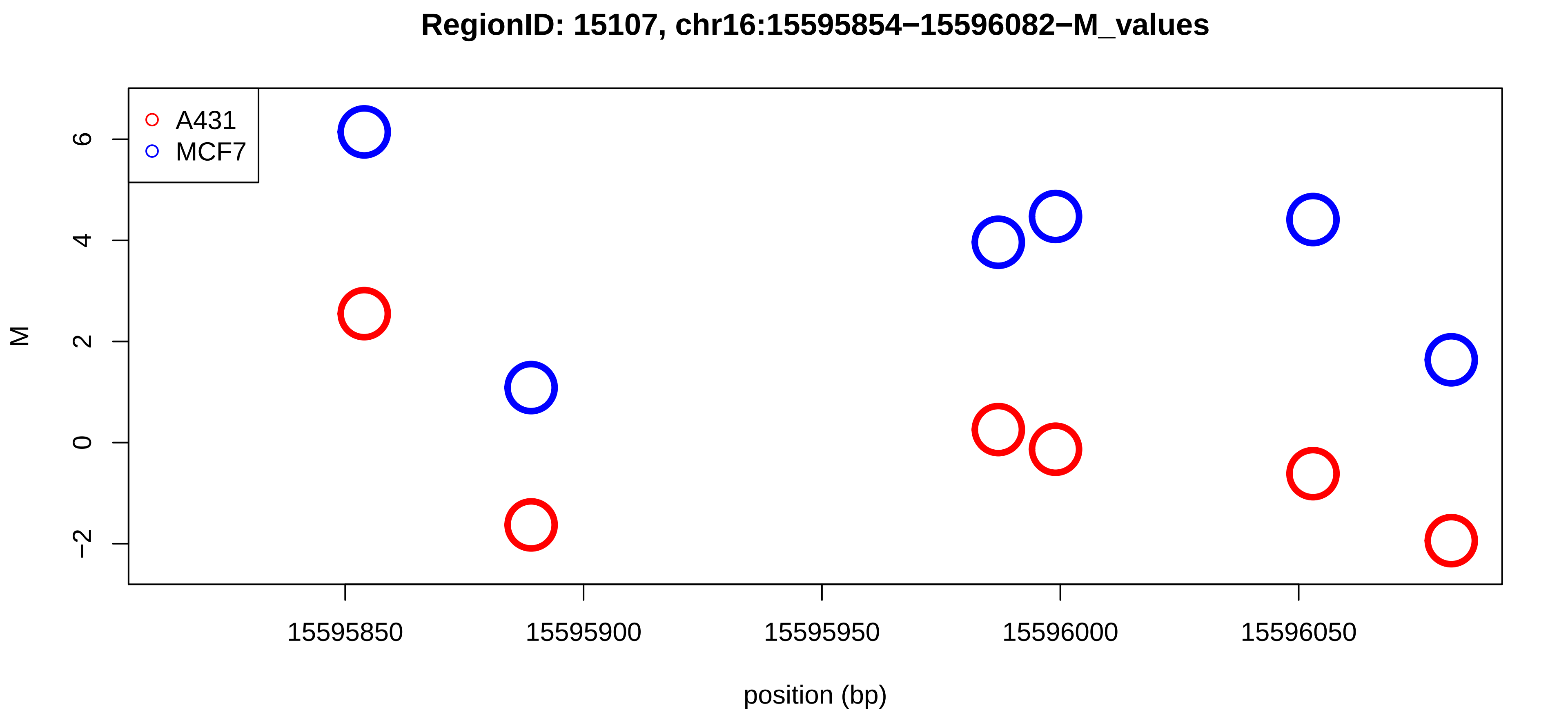
RegionID: 15107, chr16:15595854−15596082−M_values
A431
MCF7
M
−2
0
2
4
6
15595850
15595900
15595950
15596000
15596050
position (bp)

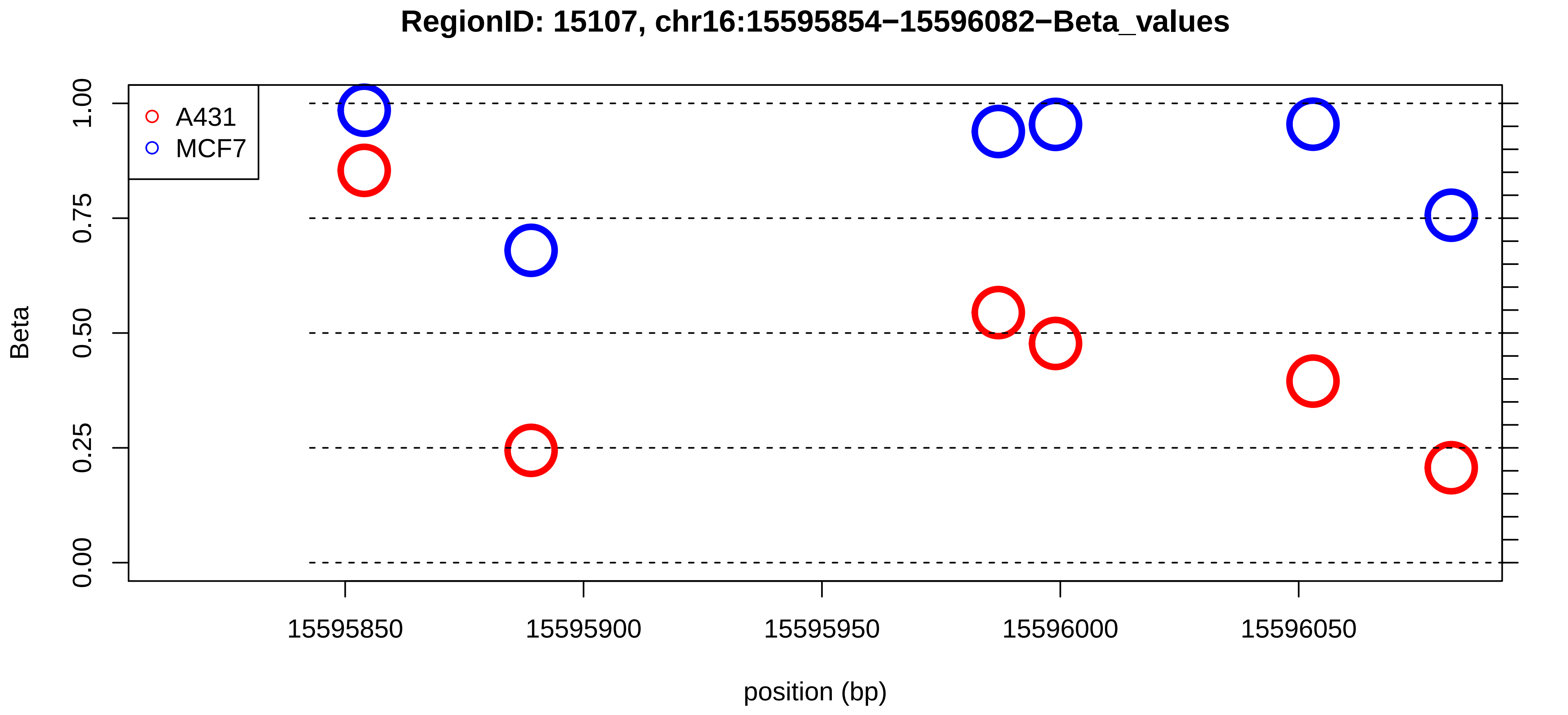
RegionID: 15107, chr16:15595854−15596082−Beta_values
A431
MCF7
Beta
0.00
0.25
0.50
0.75
1.00
15595850
15595900
15595950
15596000
15596050
position (bp)

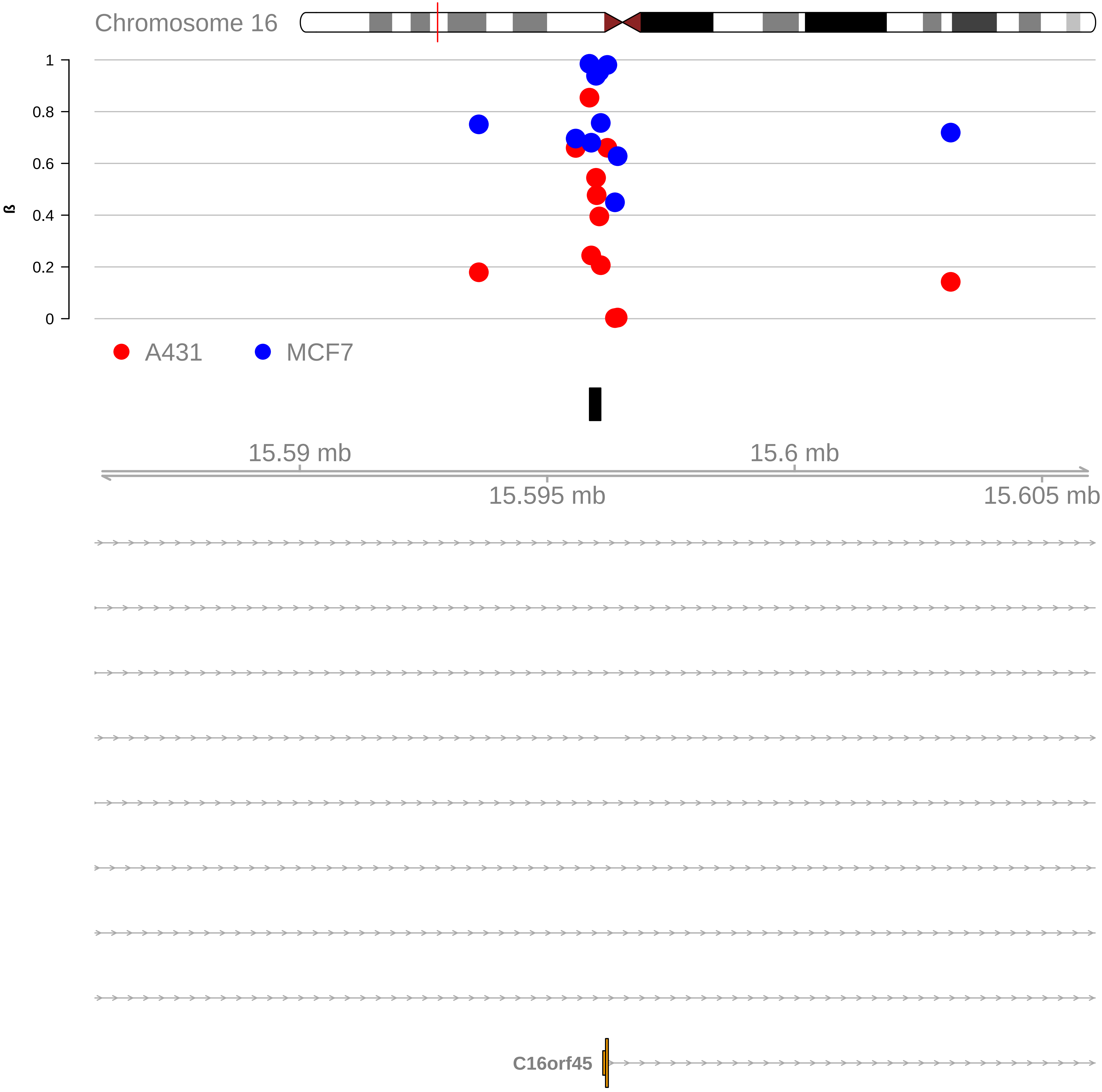

Chromosome 16
1
0.8
0.6
0.4
0.2
0
ß
A431
MCF7
15.59 mb
15.6 mb
15.595 mb
15.605 mb
C16orf45
